# Effect of electric currents on antibacterial effect of chlorhexidine against *Entrococcus faecalis* biofilm: An *in vitro* study

**DOI:** 10.4317/jced.55369

**Published:** 2018-12-01

**Authors:** Mohammad Froughreyhani, Amin Salemmilani, Aysan Mozafari, Mohammad Hosein-Soroush

**Affiliations:** 1 Associate Professor. Department of Endodontics, Dental and Periodontal Research Center, Faculty of Dentistry, Tabriz University of Medical Sciences; 2 Post-graduate Student. Department of Endodontics, Dental and Periodontal Research Center, Faculty of Dentistry, Tabriz University of Medical Sciences

## Abstract

**Background:**

This *in vitro* study was mainly aimed to evaluate the effect of high-frequency alternating currents (AC) applied by an electronic apex locator (EAL) on the antibacterial properties of chlorhexidine (CHX) on *E. faecalis* biofilm.

**Material and Methods:**

The root canals of 120 extracted human single-rooted teeth were prepared using Gates-Glidden drills and hand K-files. After contaminating the root canals with *E. faecalis*, they were incubated for 60 days. Then, the teeth were randomly divided into six experimental groups (n=20). Group 1, 2% CHX; group 2, normal saline (NS) with direct current (DC); group 3, normal saline (NS) with high-frequency alternating current (AC); group 4, 2% CHX with DC; group 5, 2% CHX with AC; group 6, control (normal saline). The samples were collected from the root canal walls of 16 teeth in each group and 1:10 serial dilutions were prepared and added to Muller-Hinton agar (MHA) plates and incubated at 37°C for 48 h. The longitudinal sections of the other 4 teeth used to observe under a scanning electron microscope (SEM). A classic colony counting technique was used for counting the vital E. faecalis bacteria in MHA. Two-way ANOVA was used for statistical analysis of the data. The level of significance was set at *P*<0.05.

**Results:**

The electric current significantly changed the colony-forming units (CFU) values (*P*<0.001). According to pair-wise comparisons, the highest CFU difference was observed between the AC group and the group without electric current (*P*<0.001); furthermore, the difference between the DC group and the group without electric current was not significant (*P*=0.823).

**Conclusions:**

The highest bioelectric effect occurred with the use of high-frequency alternating electric current in the form of an apex locator with CHX as a canal irrigant.

** Key words:**Biofilm, Chlorhexidine, Direct current, Electric current, Enterococcus faecalis.

## Introduction

Eradication of microorganisms from the root canal system is required for a successful root canal treatment. Use of root canal irrigants along with mechanical instrumentation and intracanal medication as the standard protocol of endodontic treatment results in the elimination of the majority of microorganisms from the root canal system (1). However, despite all the efforts in this field, failures still occur due to bacterial resistence. *E. faecalis* is one of the most prevalent bacterial strains isolated from cases with failed endodontic treatment (1,2). It has been found in 24‒77% of root canals of teeth with recalcitrant periapical lesions (3,4). In addition to adaptation with the environment and resistance to antibacterial agents, *E. faecalis* is able to attach to root canal walls and produce bacterial biofilms (4-6). Biofilms show greatly increased resistance to antibiotics and antimicrobial agents (7) so that biofilm bacteria are resistant to antibacterial agents up to 5000 times (4,6) .This resistance apears to be due to their physiologic properties, including altered growth and diffusion barrier of biofilm matrix (8,9). Therefore, many studies have evaluated antibacterial effects on *E. faecalis* biofilms (10-12).

Different methods have been used to improve antimicrobial potency against the biofilm bacteria. The effect of low-intensity electric currents on destruction of bacterial biofilms was shown in 1992 (3,7) and also, it has been shown that the antibacterial effect of antimicrobial agents increased synergistacally with low-intensity electerical currents, a phenomenone called “bioelectric effect” (3,5,7). An increased antibacterial effect of tobramicyn due to the use of electrical currents against *Pseudomonas aeruginosa* has been shown in various biofilm systems (3,6,13-17). In addition, improved antibacterial effects of gentamicin against *Staphylococcus gordonii* (17) and cycloheximide against *Candida albicans* (7) have been demonstrated.

Different studies have shown the electricidal effect of electric currents on various bacteria without any antimicrobial agents (18-21) and to differentiate this phenomenon from bioelectric effect, it has been called “electricidal effect” (21). Studies have mostly used direct electric currents. Caubet *et al.* (5) used alternating high-frequency currents in their study and showed its synergism with gentamicin and oxytetracyclin against *E. coli* biofilm.

There have been no studies evaluating the effect of electric current synergism on the antibacterial properties of root canal irrigants. Chlorhexidine is one of the most common canal irrigants which unlike the others shows low toxicity, low irritation of periapical tissues, tolerable odor and taste and substantivity. Despite favorable antibacterial charactristics against planktonic bacteria (22), CHX does not affect biofilm bacteria. Furthermore, the antibacterial efficiency of CHX reduces in contact with intracanal organic materials and dentin (23,24).

Therefore, this study was carried out to evaluate the effect of antibacterial direct electric currents or high-frequency alternating currents alone or in conjunction with CHX against *E. faecalis* biofilm.

## Material and Methods

The study was approved by the Research and Ethics Committee of Tabriz University of Medical Sciences. A total of 120 (25) human single-rooted teeth extracted because of periodontal diseases were selected for this in vitro study. All the teeth had mature single straight roots, with no root caries, previous endodontic treatments or anomalies. After extraction, the teeth were stored in 3% chloramine-T solution at 4°C. An ultrasonic scaler was used to remove any calculus and remaining periodontal tissues from the root surfaces. Then the tooth crowns were eliminated with diamond disks (18) at the level of CEJ to achieve root lengths of 12 mm. The working length was determined using a #15 K-Flexofile (Dentsply Maillefer, Ballaigues, Switzerland), 1 mm away from the apical foramen. The root canals were prepared with #4, #3, #2 and #1 Gates-Glidden drills (Dentsply, Maillefer, Ballaigues, Switzerland) using the crown-down technique. For the preparation of apical thirds of the root canals, K-files (Maillefer, Dentsply) up to #50 were used. Each root canal was irrigated with 3 mL of 5.25% NaOCl (Taj Corp, Tehran, IRI) between each file size. Finally, 1 mL of 17% ethylenediaminetetraacetic acid (EDTA, Pulpdent Corp, Watertown, MA, USA) was used for 3 min to remove the smear layer. The teeth were sterilized in an autoclave at 121°C and 15 psi pressure for 20 min. To confirm the sterility of the teeth, they were incubated for 24 hours at 37°C in brain-heart infusion broth (BHI, Merck, Darmstadt, Germany).

-*E. faecalis* Biofilm Preparation

The teeth were passed through the holes created on the plastic caps of sterile penicillin vials containing BHI broth so that the apical ends of the teeth were placed within BHI.

A standard suspension of *E. faecalis* (ATCC 29212, Reference Laboratories of Iran Research Center, Tehran, Iran) was prepared in the Bacteriology Laboratory of Tabriz Faculty of 

Medicine. The microorganisms were incubated for 24 hours in solid brain-heart infusion (BHI, Merck, Darmstadt, Germany) at 37°C and a final concentration of 3×108 cell/mL (1 McFarland turbidity) was prepared. Then 5 mL of bacterial suspension was mixed with 5 mL of sterile BHI broth and inoculated within the canals with a sterile micropipette. This procedure continued every 48 hours up to 60 days by placement of a fresh bacterial culture (1 McFarland turbidity) into the canals. During this time, the teeth were incubated at 37°C (26).

-Antibacterial Protocols

After 60 days, the teeth were randomly divided into six experimental groups (n=20). Group 1, 2% CHX; group 2, normal saline (NS) with direct current (DC); group 3, normal saline (NS) with high-frequency alternating current (22); group 4, 2% CHX with DC; group 5, 2% CHX with AC; group 6, control (normal saline). In group 1, the root canals were irrigated with 1 mL of 2% CHX solution (F.G.M., Joinville, Brazil), which remained in the canals for 5 min and then the canals were irrigated with 3 mL of sterile normal saline solution. In group 2, the root canals were irrigated with 1 mL of normal saline solution, and K-file #50 which served as a cathode was placed up to the working length (WL) and connected to an electrical device that generated 0.6 mA DC (a current density of 6 mA/cm2) and the other wire that served as an anode was placed in the PBS and DC was applied for 5 min. Then the canals were irrigated with 3 mL of sterile normal saline solution. In group 3, the root canals were irrigated with 1 mL of normal saline solution; then one K-file #50 was placed up to the WL and connected to Root-Zx (J. Morita Corp, Kyoto, Japan) electronic apex locator that generated AC and the lip clip was inserted in the PBS and it was activated for 5 min. Then the canals were irrigated with 3 mL of sterile normal saline solution. In group 4, after irrigation with 2% CHX, DC was applied for 5 min similar to that in group 2. Then the root canals were irrigated with 3 mL of sterile normal saline solution. In group 5, after irrigation with 2% CHX, Root-Zx (J. Morita Corp, Kyoto, Japan) electronic apex locator was activated for 5 min similar to that in group 3; then the root canals were irrigated with 3 mL of sterile normal saline solution. In all the experiments, the flow of the electric current was confirmed by placing an ammeter.

**Figure 1:**
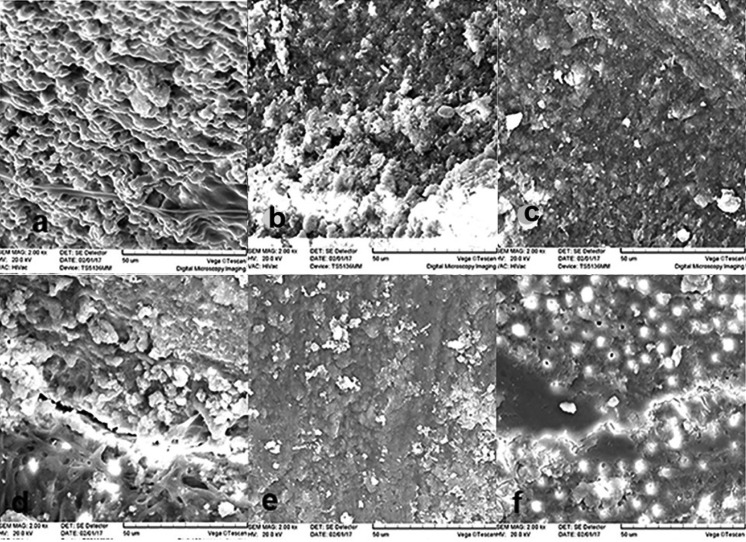
a) Following normal saline (NS) irrigation, thick biofilm was still observed on the surface of the canal wall. b) After adding Direct curent (DC) to NS irrigation, the canal wall still contained a large number of bacteria. So the application of DC alone didn’t have much effect on biofilm bacteria. c) Presence of high-frequency alternating current (AC) in NS irrigation killed much biofilm bacteria although it was a little but the reduction was significant compared with a and b. d) Following Chlorhexidine (CHX) irrigation, the number of surviving bacteria was reduced by application of CHX compared with NS. e) After adding Dc to CHX irrigation, the spread of the biofilm was evenly over the entire wall and a thin bacterial biofilm was remained on the surface of the canal wall. f) Presence of AC in CHX irrigation was completely effective in removing biofilm from the surface of the canal wall.

After antimicrobial protocols, 4 teeth from each group were placed in 2.5% glutaraldehyde for 7 days and longitudinal sections were prepared for the observation of biofilms under a scanning electron microscope (Camscan MV 2300, Czech Republic) (Fig. 1).

Other roots were stored at -25°C for 24 h to prevent or reduce generation of heat during the sampling phase. Dentin chips produced as a result of drilling the root canal walls with #5 and #6 Gates-Glidden drills were collected in order to evaluate disinfection efficacy. The drills were placed in the root canals up to 1 mm short of the WL and approximately 10 µg of dentin chips were collected from each root canal, which were carried into sterile tubes that contained 2 mL of normal saline solution, followed by vortexing for 20 sec. Serial one-tenth dilutions were prepared. Then 1 mL of each solution was added to 3 Muller-Hinton agar plates, followed by incubation at 37°C for 48 h. All the procedures were carried out by observing aseptic conditions in a luminar flow chamber with sterile instruments. A classic colony counting procedure was applied to determine vital *E. faecalis* bacterial counts in Muller Hinton agar plates. After determining the colony-forming unit (CFU) in each root canal, the mean CFU of each group was determined ( Table 1).

**Table 1:**
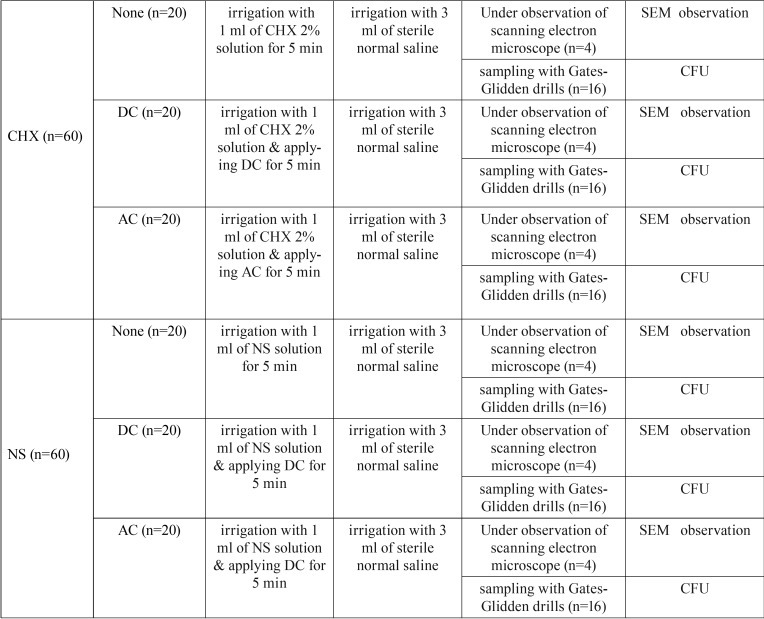
The distribution of groups.

-Statistical Analysis

Statistical analysis was performed with SPSS (Statistical Package for Social Science, SPSS, Version 20.0, SPSS, Chicago, IL, USA).

 Table 2 presents the colony counts (CFUs) in six experimental groups for different irrigants and electric currents. Quantitative data were reported as means, medians, 95% confidence interval, maximums and minimums.

**Table 2:**
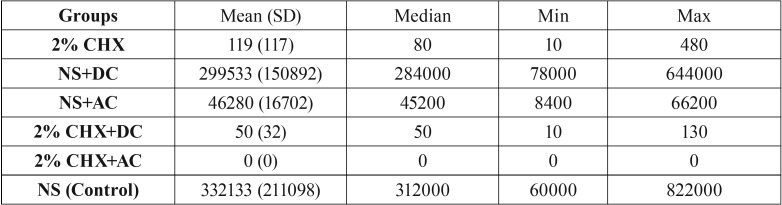
The CFUs in 6 study groups.

Two-way ANOVA was used to compare the mean CFU values in groups of DC, AC and without current, regardless of the irrigants.

The results of Kolmogorov-Smirnov test showed normal distribution of data only in the normal saline solution group. One-way ANOVA was used for inter-group analysis in NS groups, and the HSD Tukey test was used for pair-wise comparisons; Kruskal-Wallis test was used for inter-group analysis in CHX groups; Mann-Whitney U test was used for pair-wise comparisons ( Table 3). The level of significance was set at *P*<0.05.

**Table 3:**
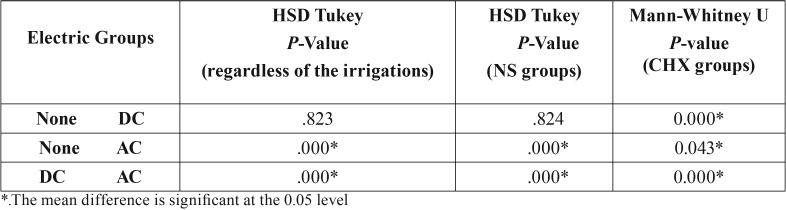
Pair-wise comparisons of the CFUs.

## Results

The results of two-way ANOVA showed that electric current significantly changed the CFU values (*P*<0.001). According to pair-wise comparisons, there were significant differences between the AC and DC groups and also the AC group and the group without electric current (*P*<0.001), but the difference between the DC group and the group without electric current was not significant (*P*=0.823). The highest CFU difference was observed between the AC group and the group without electric current, with 142986.33 units higher in the group without current. Also in the NS groups, according to pair-wise comparisons, there were significant differences between the AC and DC groups and also between the AC group and the group without electric current (*P*<0.001), but the difference between the DC group and the group without electric current was not significant (*P*=0.824). In addition, in CHX groups, according to pair-wise comparisons, there were significant differences between the AC and DC groups and the AC group and the group without electric current and also between the DC group and the group without electric current.

## Discussion

This study evaluated the effect of electric currents on antibacterial efficacy of chlorhexidine as a root canal irrigant against *E. faecalis* biofilm. The rationale for the selection of *E. faecalis* in the present study was that it is one of the most resistant intracanal bacteria and also it could create extra- and intra-radicular biofilms which are very difficult to eliminate from the infected root canals (10-12).

Electric currents improve the efficiency of biocides and antibiotics in destroying bacterial biofilms (21), and it has been suggested that electric currents could be considered as a safe and inexpensive method to increase the entrance of different therapeutic agents into the cells (27). In addition, electrostatic forces between bacterial species and surfaces are usually repulsive because virtually all the biomaterials are negatively charged similar to bacteria. It has been suggested electric currents might enhance such forces, resulting in the detachment of bacterial biofilms from the surface (17-20). In this study, two types of electric currents, including direct and alternating with high frequency applied by an electronic apex locator, were used. The results showed that the electric current, irrespective of the type of the irrigation agent, resulted in a decrease in bacterial colony counts. Consistent with our results, Liu *et al.* (19), Del Pozo *et al.* (18), and Stoodley *et al.* (28) concluded that corrosion occurred in metal electrodes used for evaluation of the effect of electric currents on the bacteria and the effect of the antibacterial activity of electric currents. Furthermore, it has been suggested that products of electrolysis increase the bactericidal efficiency of electric currents by increasing the amount of ions on the surface or within bacterial cells.

Moreover, the results of this study showed that the effect of direct current was significantly different between the groups in terms of the type of the irrigation agent. The use of DC in the 2% CHX group resulted in a decrease in bacterial colonies, which was significantly more than that in the 2% CHX group without electric current. Although, the effect of direct current in the normal saline group as a canal irrigant reduced the colony counts, there was no significant difference from the normal saline group without electric current. Several ex vivo studies have reported similar results on the use of electric DC (4,13,14) and declared that bioelectric effect could eradicate different gram-positive and gram-negative species and fungi (6).

Furthermore, consistent with the results of this study, Wellman *et al.* (17) confirmed that electric current alone had no or minimal effect on bacterial biofilm so strilant plus electric currents were necessary to achieve the optimum level. Furthermore, Wellman *et al.* (17) showed that there was a dose-dependent response in combining electric currents with antibiotics. Moreover, Jass *et al.* (14) concluded that electric currents could only improve the activity of some antibiotics. Therefore, the type of the material applied with electric current is important in decreasing bacterial counts. CHX has antibacterial effects without solubility effect; however, its application in association with electric current with low intensity (DC) could reduce the colony counts more than CHX alone. Therefore, application of electric currents increases its bioelectric effect and might cause solubility effect but have a minimal effect with normal saline. In addition, the results of the present study showed that the type of the material was effective up to 55% in decreasing bacterial counts.

In addition, the results of this study showed that the effect of alternating current (22) applied by an electronic apex locator in the normal saline group, and in particular in the CHX group resulted in the highest decrease in bacterial counts, which might be attributed to the higher electric current intensity in the electronic apex locator in comparison to the direct current. Also Stoodley *et al.* (28) showed that structural changes of biofilms, referred to as electrostatic influence, could occur due to the interaction between groups with charge in biofilm, pH effect and wire (electrode) charge, declaring that expansion and shrinkage of bacterial biofilm on the electrode surface occurred due to current polarity reversal.

However, in the present study, pH changes around the electrods and biofilm and the rate of structeral changes during the application of electric current were not mesured and further studies are necessary to evaluate pH changes on bacterial biofilms. Moreovere, further studies are recommended to evaluate the effect of electric currents with different intensities and on various bacterial species. However, the exact mechanism of the antibiofilm activity of DC and AC is unclear, necessitating further investigations.

## Conclusions

According to the results of this study, application of electric current irrespective of type of canal irrigant could be effective in decreasing bacterial counts. The highest bioelectric effect was demonstrated when high-frequency alternating electric current was applied by an electronic apex locator with CHX as a canal irrigant.
